# Scraps

**Published:** 1887-12

**Authors:** 


### SCRAPS

PICKED UP BY THE ASSISTANT-EDITOR.

---

**Examinations.**—In a story in *The Hospital* it is said that an examiner, failing to get from a candidate answers to several questions in succession, handed him a visiting card and said to him, "Mr. Brown, will you be kind enough to put down on that card all you *do* know of the science of medicine."

**Too many at once.**—In reference to the Latin epigram which appeared in the September number, a subscriber writes: "I send you the product of a wakeful night. Not very elegant, but literal. They ought to be elegiac hexameters and pentameters. But that was rather too hard a job."

I was rather below par, but you quickly found me out,
O Symmachus, and came, and your hundred pupils too;
With their hundred icy fingers they pulled me all about:
Before, I felt no fever; now, Symmachus, I do.

IDEM GRÆCE.

Ἄρρωστος ἦν· ἀλλ' εὐθέως προσέρχεται
Ἕκατον μαθήταις Σύμμαχος κυκλοῦμενος·
Ψύχροι τε μ' ἕκατον ψηλαφῶσι δάκτυλοι
Οὐκ εἴχον, ὤμοι, πύρετον, ἀλλὰ νῦν ἔχω.

**Recent Therapeutics.**—*The Medical World* perpetrates the following, in reply to a question as to the value of the Bergeon treatment of consumption: "It certainly detracts the mind of the patient for a while, and may do good by its moral effect, by making him think of his latter end. He becomes, as it were, conscious of his rectum, or, as the schoolboy renders it, *mens conscia recti*. He may even 'bacilli' enough to cherish the hope that he is being cured, and is taking a short cut to health over his *pons asinorum*, but we regret to say that he will find the road to end, as it begun—in gas."—*The Medical Age*.

As an instance of the danger of being funny, *The Medical Record* says: "The humourous *feuilletoniste* of the *Union Mèdicale* not long ago argued that no apparatus was necessary for carrying out the Bergeon method of treating consumption. It was only necessary to make the patient eat beans. He now states that Professor Ewald afterward presented the idea, in all seriousness, at a meeting of the *Gesellschaft für innere Medicin* of Berlin."

**Re-Vaccination.**—The following letter will, during the present small-pox scare, be read with great interest: "New Street, Hanover Square, July 7th, 1817. Sir,—Agreeably to your request, I transmit to you the result of my practice in vaccination. I have vaccinated more than twelve thousand persons, many of whom have been put to the test of variolous matter, both by inoculation and exposure to the infection in the natural way. It has been long and satisfactorily ascertained that vaccination affords as complete a security against the future infection of the small-pox as variolous inoculation. There are certainly exceptions of both kinds. Instances have occurred in which small-pox has appeared both after small-pock and cow-pock inoculation, probably in an equal proportion; but there is this remarkable difference, that when it takes place after the cow-pock, it is commonly in a mitigated form. I have long recommended a second vaccination, or even a more frequent repetition of the process, in order to remove any scruples that may be entertained on the subject, but must report my decided conviction that those persons who have had the cowpock are full as safe as those who have had the small-pox. Several instances have occurred in which small-pox has taken place a second time, and proved fatal. One occurred near Newbury, and another at Chichester, both of which proved fatal. It is, perhaps, unnecessary to observe that when I re-vaccinate any person, I insist on doing it gratuitously, and make it a point to inoculate with two or three contiguous punctures in each arm with active virus in a fluid state. If I can communicate any further information that may be useful to the public or satisfactory to yourself, you may at all times command my services. I remain, with great respect, sir, your most obedient humble servant, JOHN RING. To Dr. LEWIS, Bath."

Mr. Augustin Prichard, in sending the above, says: "I have lately fallen in with this letter, written from a London practitioner to a great-uncle of mine, Dr. Lewis, who practised at Bath at the beginning of this century; and as the perennial question of the advantage or the necessity of re-vaccination is once more cropping up, the opinion of an early vaccinator may be of interest; and it must be remembered that they had then always virulent small-pox to deal with and to test their success. There is one paragraph with which your readers will scarcely agree."

**Science in Court.**—A German trade journal relates a curious instance in which a legal decision was arrived at by means of a scientific experiment in a court of law. One of the workmen employed by a large firm let his hammer slip from his fingers just as it was descending upon the object on which he was employed. It struck another workman in the region of the left eye, and produced a serious wound. The sufferer was attended by a surgeon, and in a short time after the accident all trace of its results had disappeared. The patient, however, persisted in declaring that he was enduring great pain, and that he had completely lost the sight of his left eye. The head of the firm, who was legally responsible for the consequences of the occurrence, employed several specialists, who met in consultation, and who could discover nothing wrong with the eye. Compensation was therefore refused to the man, and the matter was taken into court by him. Here, again, the experts commissioned by the judge to examine the eye unanimously declared that it was perfectly sound and uninjured. The plaintiff persisting that he could see nothing with the left eye, one of the experts had recourse to the following experiment. He took a black board and wrote some words upon it in green ink. Then he desired the plaintiff to put on a pair of spectacles which had been specially prepared for this occasion. The glass for the left eye was plain white; that for the right was red. The man having adjusted them, the expert asked him to read what was written on the board, which he did without a moment's hesitation, thereby convicting himself of fraud, for the red glass in the right eye would turn the green ink into black, and render it quite invisible upon the black board. He had read the writing with his left eye only, the spectacles on that side having an ordinary white glass The result of the experiment was the nonsuiting of the plaintiff, who was besides condemned to pay the costs of the action. The application of this novel form of science to his case must have been extremely surprising to the workman, who in his ignorance was doubtless firmly convinced that no one in the world could be in a position to contradict his assertion about his own left eye. His position was, to his own thinking, impregnable; and to be found out by a bit of jugglery with a pair of spectacles and a black board must have been all the more exasperating because it was so absolutely unexpected.—*The Daily News.*

[Mr. Cross tells me that this well-known test of Snellen's is being frequently applied. The difficulty rests in getting the exact shade of green which will be invisible through a red glass. The case quoted is a good instance of the practical value of the test.]

